# The Versatile Vascularized Second Metacarpal-Base Bone Graft

**DOI:** 10.3390/jcm13144214

**Published:** 2024-07-19

**Authors:** Thierry Christen, Célina Cottier, Sébastien Durand

**Affiliations:** Department of Plastic and Hand Surgery, Centre Hospitalier Universitaire Vaudois, 1005 Lausanne, Switzerland; thierry.christen@chuv.ch (T.C.); celina.cottier@hopitalvs.ch (C.C.)

**Keywords:** vascularized bone flap, second metacarpal, nonunion, dorsal metacarpal artery

## Abstract

Vascularized bone grafts of the wrist and hand help to achieve bone union in various clinical settings such as bone fusion or nonunion treatment. Among the multiple possible donor sites, the base of the second metacarpal is convenient because of its relatively straightforward harvesting, lack of impairment of the hand vascularization, sufficient bone supply for small joint fusion and phalanx or metacarpal nonunion management. The flap is versatile as it can reach any carpal and metacarpal bone as well as the proximal phalanx of all fingers. The arc of rotation of the flap depends on which side of the dorsal intercarpal arch it is based, either radially or ulnarly. It can also be pedicled distally by a retrograde flow through the second dorsal metacarpal artery. The robust vascularization of the flap allows for reliable healing. We present five new indications in various clinical settings that illustrate the versatility of the arc of rotation of the flap.

## 1. Introduction

Bone grafting stimulates the healing process and is often used to increase fusion rates. Vascularized bone grafting is technically more challenging but could offer significant advantages such as faster healing and reduced bone resorption due to the additional blood supply and the maintenance of the cell viability [1]. The donor sites of free vascularized bone graft include the fibula, iliac crest, metatarsal, medial femoral condyle [2], rib, humerus, ulna and scapula, but these procedures require microsurgery, an additional exposure for flap harvest and are not without donor-site morbidities.

Various local pedicled bone flaps are available in the wrist, but the reach of these flaps is often limited because of a short pedicle length, which restricts their indications. Multiple donor sites have been reported such as the dorsal distal radius [3,4], the palmar distal radius [5,6], the first metacarpal [7] and the pisiform [8]. For example, in patients with chronic nonunion of the scaphoid, a vascularized bone graft from the distal dorso-radial radius or taken from the medial part of the radial epiphysis [3,5] can be easily harvested and permits bone fusion and good clinical results.

An ideal donor site has the following important conditions: appropriate quantity of bone, sufficient length of the flap pedicle, constant pedicle anatomy without variations, low donor-site morbidity, and no additional exposure for flap harvest if possible [9].

The second metacarpal base can also provide a vascularized bone flap [10]. It has been reported for various indications including scaphoid nonunion and Kienböck’s disease [9], idiopathic osteonecrosis of the capitate [11] or to obtain fusion of the trapeziometacarpal joint [12] and for reconstruction of bone defects of the distal phalanx and distal interphalangeal joint [13,14].

**Figure 1:**
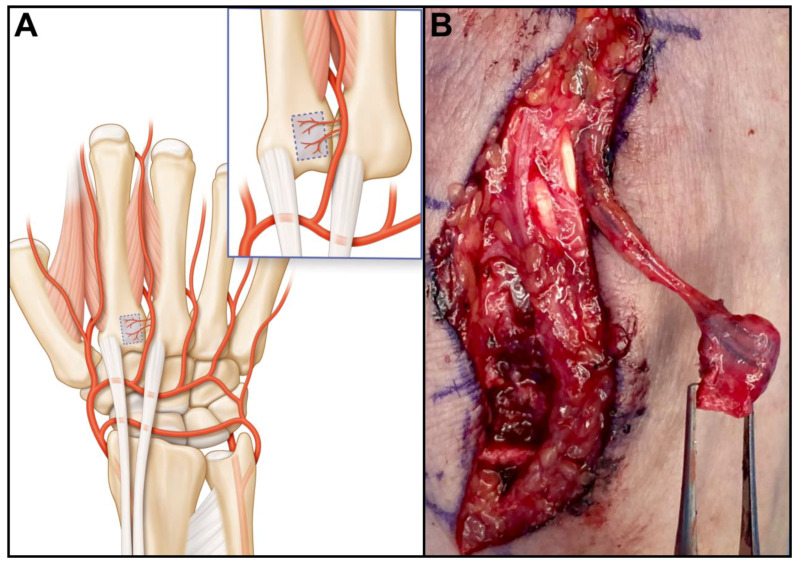
Vascularization of the dorsal aspect of the wrist. For simplification, only the dorsal radio-carpal and intercarpal arches are represented, the thin basal metacarpal arch has been omitted (**A**). The second dorsal metacarpal artery provides thin periosteal vessels to the base of the second metacarpal; (**B**) Operative image of a 2DMA bone graft pedicled distally.

The anatomy of the dorsal carpal arterial network of the carpus is composed of the radiocarpal, the intercarpal and the basal metacarpal arches [15]. These arches are connected to the radial, ulnar, posterior and anterior interosseous arteries. The second dorsal metacarpal artery (2DMA) consistently provides vascularization of the base of the second metacarpal (Figure 1A) through small periosteal vessels. The vessel diameter in the donor region of a vascularized bone graft of the second metacarpal bone was approximately 58 μm. [16]. The mean diameter of the 2DMA is 1.3 mm [16].

The flap pedicle is either retrograde through the 2DMA (Figure 1B) or antegrade on the radial or ulnar aspect of the dorsal intercarpal arch. The versatility of the vascularization provides a wide arc of rotation of the flap, its indications being multiple. The purpose of this study is to illustrate the versatility of the flap with five new indications in various clinical situations and report on its reliability relative to the achievement of bone union.

## 2. Materials and Methods

Clinical cases: The present investigation consists in a retrospective analysis on the institutional database of a single hospital. This manuscript was reported following the STROBE guidelines. The study was performed in accordance with the Helsinki Convention. Ethical approval was not required by our Ethics Committee for reporting human case series (< or =5). Informed consent was obtained from all subjects involved in the study. Written informed consent has been obtained from the patient(s) to publish this paper. A search of our institutional database was performed to identify all patients who had undergone second metacarpal-base bone graft between 1 January 2016 and 31 January 2024. We present five cases (performed by the same surgeon (S.D.)) in new and various clinical settings (inclusion criteria) that illustrate the versatility of the arc of rotation of the flap. Bone fusion and clinical assessment are reported for each clinical case.

**Figure 2:**
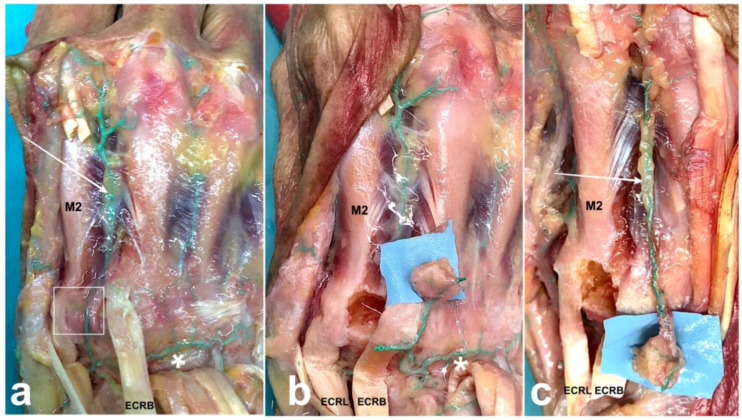
Surgical technique of vascularized second metacarpal-base bone graft. Antegrade injection of green latex was injected into the brachial artery of fresh cadaver. Skin and extensor digitorum communis excisions were made on the dorsal aspect of the right hand. Note the small branches to the ulnar border of the second metacarpal bone base (white square) coming from the 2DMA (white arrow). 2DMA arises from the dorsal carpal arch (white asterisk) (**a**). When the recipient site is proximal and ulnar to the second metacarpal base, distal ligation of the 2DMA and division of the radial side of the dorsal carpal arch are required (**b**). When the recipient site is distal to the metacarpal base, the 2DMA is ligated proximally to the periosteal branches (**c**). ECRB: extensor carpi radialis brevis; ECRL: extensor carpi radialis longus. M2: second metacarpal bone.

Operative procedure: Under tourniquet with no exsanguination, a longitudinal, oblique or transverse incision was made according to the location of the recipient site. The dorsal carpal arch and the 2DMA are identified. Care should be paid to make sure the 2DMA arises from the dorsal carpal arch and not solely from a palmar perforating branch if the recipient site is proximal to the metacarpal base. In some cases, the 2DMA might have an intramuscular course through the second dorsal interosseous muscle but remains connected to the dorsal carpal arch proximally. The tendon of the extensor carpi radialis longus is retracted to clear the ulnar half of the base of the second metacarpal, which is inspected for periosteal branches coming from the 2DMA (Figure 2a). The second carpometacarpal joint is delineated with hypodermic needles under fluoroscopy prior to choosing the flap location. The borders of the flap are drilled with a 1 mm k-wire in a stamp-like manner to weaken the cortical bone. A small osteotome allows completing the osteotomy and lifting the flap while paying attention to keep the cancellous bone attached to the cortex. 

The choice of the pedicle is based on the location of the recipient site. Distal ligation and division of the 2DMA are required when the recipient site is proximal to the second metacarpal base. Further division of the ulnar side of the dorsal carpal arch allows the graft to reach the radial side of the wrist with a mean pedicle length of 3.3 ± 0.3 cm [16]. 

A radial division is required for ulnar transposition (Figure 2b). In this case, the arterial dissection is performed under the extensor tendons of the second to fourth rays. The graft should be pedicled on a single vessel for a maximal arc of rotation; this often requires dividing several arterial branches of the dorsal carpal arch.

When the recipient site is distal to the metacarpal base, the 2DMA is ligated proximally to the periosteal branches (Figure 2c). The dissection of the 2DMA is always possible down to the perforating branch from the deep palmar artery at the level of the metacarpal neck. A more distal dissection has been described using the vascular connection between 2DMA and the palmar digital artery through the dorsal arterial network at the level of the proximal phalanx [13,14]. For the index finger, it relies on the small diameter ulnar dorsal digital artery [15]. The donor site is filled with cancellous bone allograft (Allobone, Neutromedics, Switzerland). The tourniquet is released to check for graft vascularization, and punctuate bleeding should be seen. After preparation of the recipient site, the graft is transposed and maintained with a plate, screws or k-wires. Skin is sutured, and a splint is applied to avoid stretching the pedicle or displacement of the graft.

## 3. Results

**Figure 3:**
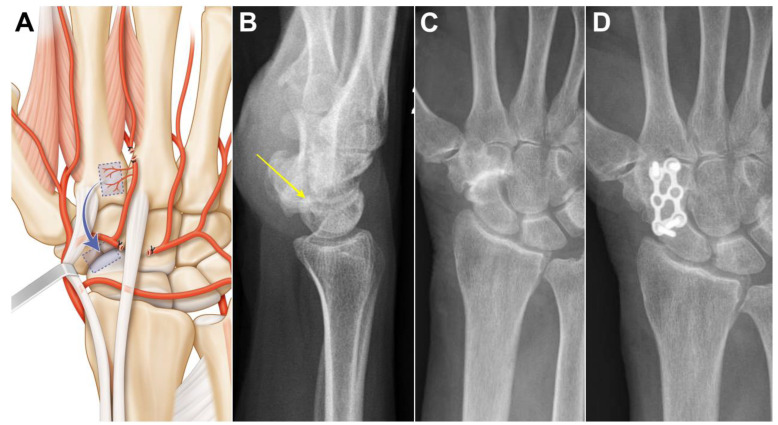
Case 1. (**A**) Arterial anatomy, the radially based pedicle necessitates ligating distal and ulnar branches; (**B**,**C**) preoperative X-ray showing advanced scaphotrapeziotrapezoidal arthritis and no DISI (yellow arrow); (**D**) union is obtained in less than three months.

Case 1: A 73-year-old female suffering from long-standing left scaphotrapeziotrapezoidal arthritis. Surgery was proposed after failure of conservative treatment. The patient underwent a scaphotrapeziotrapezoidal bone fusion. A longitudinal incision was made in line with the 2nd metacarpal ray over the carpus. Anastomosis between the dorsal carpal arch and the 2DMA was verified, and the 2DMA was ligated distally to the base of the second metacarpal (Figure 3A). Bone, periosteum and 2DMA were harvested and proximally transferred. The size of the pedicled bone was 1.0 × 0.9 cm. The dorsal carpal arch was cut ulnarly to the 2DMA origin and released up to its origin at the radial artery for STT fusion. The 2DMA bone flap was pedicled on the radial side of the dorsal intercarpal arch and fixed with a plate and 2.0 mm screws (Aptus, Medartis, Switzerland). Union was obtained after 11 weeks (Figure 3B,C). No complications were observed at the donor and recipient sites. Clinical and radiological outcomes were unchanged 2 years after surgery.

**Figure 4:**
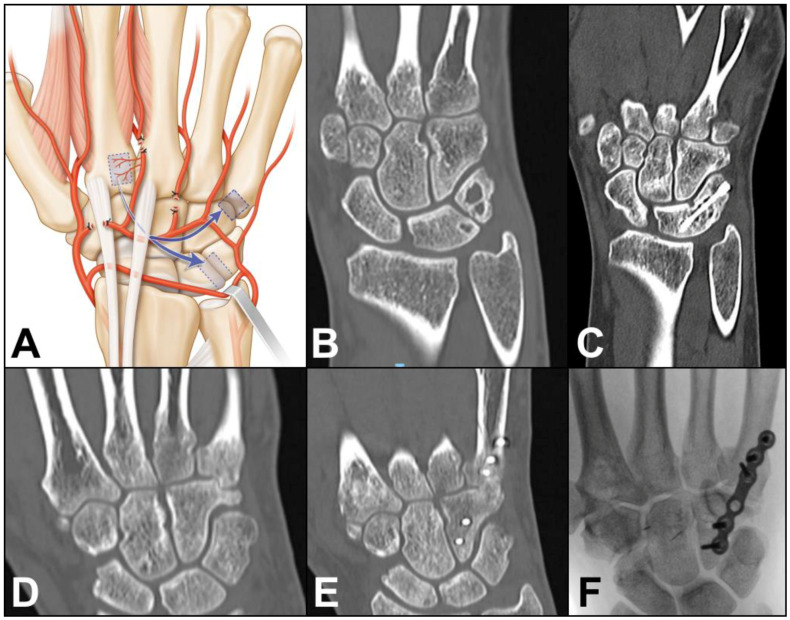
(**A**) Arterial anatomy, ligation of several arterial branches is necessary to allow the graft to reach the ulnar side of the carpus; Case 2. (**B**) preoperative CT-scan showing lunotriquetral arthritis; (**C**) postoperative CT-scan confirming fusion of the joint. Case 3. (**D**) preoperative CT-scan showing fifth carpometacarpal arthritis; (**E**,**F**) postoperative CT-scan and X-ray confirming fusion of the joint.

Case 2: A 30-year-old female developed painful arthritis of the right lunotriquetral joint. Surgery was proposed after failure of conservative treatment. An oblique incision was made from the base of the 2nd metacarpal to the lunotriquetral joint. Anastomosis between the dorsal carpal arch and the 2DMA was verified. The 2DMA was ligated distally to the base of the second metacarpal. The dorsal carpal arch was cut radially to the 2DMA origin and released up to the origin of the fourth dorsal metacarpal artery (Figure 4A). The arterial dissection is performed under the extensor tendons of the second to fourth rays. The size of the pedicled bone was 0.9 × 0.9 cm. The 2DMA flap was based on the ulnar side of the dorsal intercarpal arch. Fusion of the affected joint was achieved with a 3.0 mm CCS screw (Aptus, Medartis, Switzerland). A CT-scan confirmed bone union after twelve weeks (Figure 4B,C). No complication was observed at the donor site, and the CCS screw was removed after one year. Clinical and radiological outcomes were unchanged 2 years after surgery.

Case 3: A 39-year-old male developed painful arthritis of the fifth carpometacarpal joint (Figure 4D). Surgery was proposed after failure of conservative treatment. A transverse incision was made from the base of the 2nd metacarpal to the base of the 5th metacarpal. Anastomosis between the dorsal carpal arch and the 2DMA was verified. The 2DMA was ligated distally to the base of the second metacarpal. The dorsal carpal arch was cut radially to the 2DMA origin and released up to the origin of the fourth dorsal metacarpal artery (Figure 3A). The size of the pedicled bone was 0.8 × 0.8 cm. Fusion of the affected joint was achieved with a plate and 2.0 mm screws (Aptus, Medartis, Switzerland). Union was obtained after 10 weeks (Figure 4E,F). No complication was observed at the donor site, and the plate was removed after one year. Clinical and radiological outcomes were unchanged 1 year after surgery.

**Figure 5:**
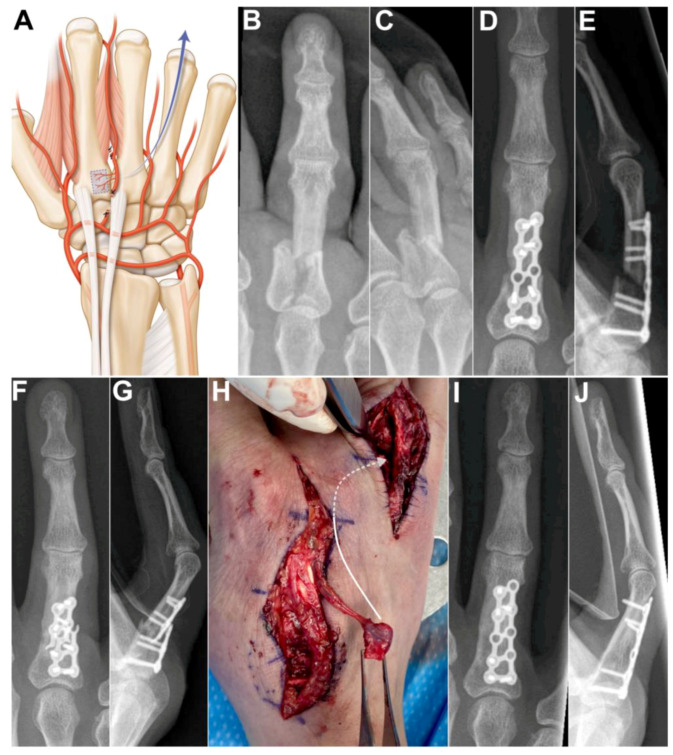
(**A**) Arterial anatomy, the distally based flap requires ligating the 2DMA proximally to the base of the second metacarpal as well as the palmar communicating branch; Case 4. (**B**,**C**) preoperative X-rays showing unstable and displaced intra-articular fracture of the first phalanx of the 4th ray. (**D**,**E**) postoperative X-rays, osteosynthesis with a dorsal plate and screws. (**F**,**G**) 7 months after osteosynthesis, X-rays showing unstable nonunion of the proximal phalanx after plate breakage; (**H**) Intraoperative photograph showing vascularized bone graft, dissection between extensor tendons and skin was made to allow passage of the vascularized 2nd metacarpal-base bone graft to the 4th ray (arrow). (**I**,**J**) postoperative X-rays demonstrating union of the phalanx.

Case 4: A 53-year-old female presented with nonunion of the ring finger proximal phalanx seven months after an osteosynthesis with a dorsal plate and screws. The absence of bone healing led to hardware failure. A longitudinal incision was made over the 2nd metacarpal and another longitudinal incision over the first phalanx of the 4th ray. After bone debridement, a 1 cm gap remained between the bone stumps. The 2DMA was ligated proximally to the base of the second metacarpal. The proximal anastomosis with the deep palmar artery was ligated (Figure 5A). The 2DMA flap was pedicled distally on the distal penetrating branch of the deep palmar artery. Gentle dissection between extensor tendons and skin was made to allow passage of the vascularized 2nd metacarpal-base bone graft to the 4th ray. The size of the pedicled bone was 0.9 × 0.9 cm. A new plate and screws (Aptus, Medartis, Switzerland) provided stability. We witnessed bone union after ten weeks (Figure 5B–E). The patient was free of pain and recovered excellent grip strength (27 kg on the right hand and 30 kg on the left hand), and the range of motion (flexion/extension) was 75/25 degrees for the metacarpophalangeal joint, 55/−10 degrees for the proximal interphalangeal joint and 50/0 degrees for the distal interphalangeal joint of the finger. No complications were observed at the donor and recipient sites. Clinical and radiological outcomes were unchanged 2 years after surgery.

**Figure 6:**
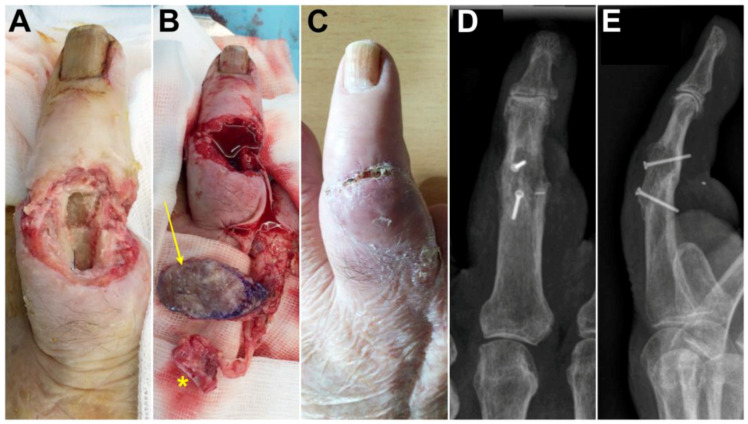
Case 5. (**A**) Photographs before surgery, dorsal aspect of the middle finger; (**B**) The elevated skin flap (arrow) and vascularized bone (asterisk); (**C**) postoperative photograph demonstrating a healed skin flap; (**D**,**E**) postoperative X-rays demonstrating fusion of the proximal interphalangeal joint.

Case 5: A 78-year-old male presented with a bone and cutaneous defect of the dorsal aspect of the middle finger 3 weeks after replantation of the middle finger (Figure 6A). A chimeric flap was designed (Figure 6B). A skin paddle supplied by the distal cutaneous branches originating from the 2DMA was associated to a second metacarpal-base bone graft vascularized by the 2DMA kept in connection with the deep palmar artery located at the level of the metacarpal head. The 2DMA was ligated proximally to the base of the second metacarpal, and the proximal anastomosis with the deep palmar artery was also ligated (Figure 5A). The size of the pedicled bone was 1.6 × 1.0 cm. The donor site was closed primarily. Although flap congestion was noted postoperatively, it disappeared on elevating the upper extremity for a week (Figure 6C). A fusion of the proximal interphalangeal joint was achieved (Figure 6D,E) with 1.5 mm screws (Aptus, Medartis, Switzerland). Bone union occurred after 3 months; the patient was free of pain and recovered contact between the pulp of the thumb and the pulp of the middle finger. Clinical and radiological outcomes were unchanged 3 years after surgery.

## 4. Discussion

The first vascularized pisiform bone graft to treat Kienböck’s disease and using the ulnar artery as a pedicle was reported in 1971 [17]. Hori et al. then successfully performed vascular pedicle implantation to necrotic bone [18]. Following these publications, transfer of a live bone graft with its nutrient vascular pedicle was introduced with good clinical outcomes in patients with long-standing nonunion or osteonecrosis. 

The base of the second metacarpal is a reliable bone flap donor site which has been reported in different clinical settings (Table 1). Although the size of the flap is limited, its wide arc of rotation provides great versatility. Previous publications have mostly focused on its use in scaphoid nonunion and Kienböck’s disease [9].

For patients with nonunion of the scaphoid with dorsiflexed intercalated segment instability (DISI), an open wedge was performed at the site of nonunion, and the bone was grafted from the volar side [19,20]. In patients without DISI, transplantation was carried out through the same dorsal skin incision. Complete bone union and DISI correction were obtained in all patients [19]. The arc of rotation of the flap in these indications is rather short; however, it can be extended by mobilizing the second dorsal metacarpal artery on the dorsal carpal arch. This requires ligating and cutting several branches either on the radial, ulnar or proximal aspect. In this way, it becomes possible to reach any intercarpal or carpometacarpal joint, even on the ulnar side, and the proximal phalanx of the digits. 

Hand surgeons should keep in mind that the origin of the 2DMA is variable. According to Khan [8], it arises from the dorsal carpal arch in 100% of 31 cadaveric specimens. Other authors report that the 2DMA is present in all dissected hands and originates from the dorsal intercarpal arch in 23% of 30 dissected hands, from a perforating branch of the deep palmar artery in 13% and is of mixed origin in 63% [21]. Vascularized second metacarpal-base graft is not possible in 13% of the cases when the recipient site is proximal to the second metacarpal base.

The 2DMA has two venae comitantes and runs along the suprafascial planes of the dorsal interosseus muscle in the second webspace, adjacent to the second metacarpal on its ulnar border and beneath the extensor indicis tendon [22]. The 2DMA gives segmental supply to overlying extensor tendons, neighboring metacarpals and underlying muscle and has a mean of six (3–12) branches. The index metacarpal receives an average of four branches on its radial side from the first dorsal metacarpal artery and three branches on its ulnar side from 2DMA [21]. The middle metacarpal receives three branches on its radial side from 2DMA [21]. The blood supply of the second metacarpal also comes from the radial artery or from the palmar metacarpal artery originating in the deep palmar arch [23]. The constant distal cutaneous branches originating from the 2DMA are located an average of 1.2 cm [24] proximally to the metacarpophalangeal joint when 2DMA reaches the distal margin of the junctura tendinae. A branch of the 2DMA, usually 3 to 4 cm upstream from the metacarpophalangeal (MCP) joint line of the index, supplies the extensor indicis proprius [25]. In our case 5, a chimeric flap was raised with the 2DMA and kept in connection with the deep palmar artery located at the level of the metacarpal head, unlike the previously cited reports in which the connection was severed [13,14]. 

Distal anastomoses between the dorsal metacarpal artery and the deep palmar artery are mostly located at the level of the metacarpal heads [21,26]. In 13% of the cases, the 2DMA presents a Y-shaped bifurcation in the proximal half of the second intermetacarpal space [21]. The 2DMA ends by two dorsal cutaneous networks [27] for the proximal phalanges of the two adjacent finger rays. Most authors noted two to four anastomoses between the dorsal artery network and the proper palmar digital artery at the level of the proximal phalanx [26,27,28]. Using the vascular connection between 2DMA and the palmar digital artery through the dorsal arterial network at the level of the proximal phalanx, a second metacarpal-base bone flap can be elevated with a pivot point at the proximal phalanx. Some authors report the use of this technique for reconstruction of bone defects of the distal index finger [13,14]. 

Other bone flaps are available on the dorsal side of the hand. For example, vascularized bone flap harvested from the third metacarpal bone was used successfully in cases with segmental defect of the proximal phalanx [29]. Anatomical studies have demonstrated that nutrient arteries penetrate the dorsum of the base of the third metacarpal bone [30], and damage of the extensor carpi radialis brevis tendon could occur during the harvesting of the third metacarpal-base bone flap. The 1,2 -intercompartmental supraretinacular arterial flap described by Zaidemberg has become a recognized pedicled bone graft. However, it is more challenging, and the pedicle has a small caliber with an average diameter of 0.3 mm, is short and does not allow reaching the more distal or ulnar carpal bones, limiting its application to a few indications only. Other pedicled alternatives, such as the volar carpal artery-based bone graft described by Kuhlmann, present similar drawbacks [4,5,31]. 

Due to their limited versatility, free vascularized bone grafts are commonly preferred. Among these, the iliac crest and medial femoral condyle are the most widely used techniques. While they offer good consolidation rates and versatility, they are technically demanding and associated with significant donor-site morbidity.

Joint fusion and nonunion treatment require bone graft. It is not clear if there is an advantage of using vascularized bone to achieve union. A systematic review including 1062 patients did not show clinical evidence that vascularized grafts lead to a greater union rate than non-vascularized bone [32]. Most of these studies report on the outcome of medium-sized groups. Some specific populations seem to benefit from a vascularized graft. Recently, Fan et al. [33] found that the 1,2 intercompartmental supraretinacular flap shows a better union rate in smokers. Since the morbidity of raising a vascularized bone graft is similar to that of harvesting non-vascularized bone, we believe it is indicated to use the former when dealing with revision surgery or when performing a bone fusion known to have a significant rate of nonunion. For instance, lunotriquetral arthrodesis has a nonunion rate up to 57% which can be responsible for persistent pain, restricted range of motion and difficulties performing activities of daily living [34]. In our case 2, the 2DMA flap was based on the ulnar side of the dorsal intercarpal arch, and fusion of the lunotriquetral joint was achieved with a screw after twelve weeks. The majority of diaphyseal nonunions of the long bones can be successfully treated by osteosynthesis and bone grafting. The main risk factors for developing a recurring nonunion are a poorly vascularized bone bed after infection, open and severely comminuted fracture, or internal fixation with large iatrogenic periosteum removal. In these cases or in our case 4, vascularized bone grafting might be justified [35,36].

Our study has some limitations, including the small sample size as well as the single-center modality and the retrospective nature, which can lead to unavoidable bias in clinical outcomes.

Surgery should aim to use techniques allowing to reproduce as closely as possible the natural healing process through biomimicry. In bone repair, this approach would call for the use of vascularized bone graft since the bone cells remain alive, and primary or secondary healing occurs as opposed to creeping substitution [37]. The periosteum through which the vessels penetrate the bone contains various cells with osteogenic potential [38]. Since cortical bone is present in the flap in addition to cancellous bone, a degree of primary stability is provided in addition to the aforementioned biologic effect.

## 5. Conclusions

We present five cases in new and various clinical settings that illustrate the versatility of the arc of rotation of the flap. The robust vascularization of the vascularized second metacarpal-base bone graft allows for reliable healing. Various local pedicled bone flaps are available in the wrist, and this flap has an appropriate quantity of bone, sufficient length of its pedicle and low donor-site morbidity.

## Figures and Tables

**Table 1:** Articles on vascularized second metacarpal-base bone flap. STT: scaphotrapezotrapezoid. CMC 5: carpometacarpal joint of the fifth ray. PIP: proximal interphalangeal joint.

Year	First Author	Indication	Size of the Bone Graft (cm × cm)	Number of Cases and Union Rate
2000	Makino [9]	Scaphoid nonunion Kienböck’s disease	1.2 × 0.8/0.8 × 0.8 1.0 × 0.5	2 (100%) 1 (?)
2003	Sawaizumi [19]	Scaphoid nonunion	?	7 (100%)
2004	Sawaizumi [20]	Scaphoid nonunion	?	14 (100%)
2014	Bermel [16]	Kienböck’s disease	0.6 × 0.6	1 (100%)
2015	Katz [14]	Bone defect of the middle third of the distal phalanx	1.0 × 0.5	1 (100%)
2018	Nakanishi [12]	Trapezio-metacarpal arthrodesis	1.2 × 1.0	3 (100%)
2020	Usami [11]	Idiopathic necrosis of the capitate	1.2 × 0.6	1 (100%)
2021	Yano [13]	Bone loss of the distal interphalangeal joint of the index finger	1.5 × 0.7	1 (100%)
2024	Current case series	STT arthrodesis	1.0 × 0.9	1 (100%)
		Lunotriquetral arthrodesis	0.9 × 0.9	1 (100%)
		CMC 5 arthrodesis	0.8 × 0.8	1 (100%)
		Bone loss of the first phalanx of the ring finger	0.9 × 0.9	1 (100%)
		PIP joint arthrodesis	1.6 × 1.0	1 (100%)

## Data Availability

All data generated or analyzed during this study are included in this article. Further enquiries can be directed to the corresponding author.
